# Therapy-Induced Neuroplasticity of Language in Chronic Post Stroke Aphasia: A Mismatch Negativity Study of (A)Grammatical and Meaningful/less Mini-Constructions

**DOI:** 10.3389/fnhum.2016.00669

**Published:** 2017-01-06

**Authors:** Guglielmo Lucchese, Friedemann Pulvermüller, Benjamin Stahl, Felix R. Dreyer, Bettina Mohr

**Affiliations:** ^1^Brain Language Laboratory, Department of Philosophy and HumanitiesFreie Universität Berlin, Berlin Germany; ^2^Berlin School of Mind and Brain, Humboldt-Universität zu BerlinBerlin, Germany; ^3^Department of Neurology, Charité Universitätsmedizin Berlin, Campus MitteBerlin, Germany; ^4^Max Planck Institute for Human Cognitive and Brain SciencesLeipzig, Germany; ^5^Department of Psychiatry, Charité Universitätsmedizin BerlinCampus Benjamin Franklin, Berlin Germany

**Keywords:** cortical reorganization, aphasia, syntax, EEG, intensive language therapy, mismatch negativity

## Abstract

Clinical language performance and neurophysiological correlates of language processing were measured before and after intensive language therapy in patients with chronic (time post stroke >1 year) post stroke aphasia (PSA). As event-related potential (ERP) measure, the mismatch negativity (MMN) was recorded in a distracted oddball paradigm to short spoken sentences. Critical ‘deviant’ sentence stimuli where either well-formed and meaningful, or syntactically, or lexico-semantically incorrect. After 4 weeks of speech-language therapy (SLT) delivered with high intensity (10.5 h per week), clinical language assessment with the Aachen Aphasia Test battery demonstrated significant linguistic improvements, which were accompanied by enhanced MMN responses. More specifically, MMN amplitudes to grammatically correct and meaningful mini-constructions and to ‘jabberwocky’ sentences containing a pseudoword significantly increased after therapy. However, no therapy-related changes in MMN responses to syntactically incorrect strings including agreement violations were observed. While MMN increases to well-formed meaningful strings can be explained both at the word and construction levels, the neuroplastic change seen for ‘jabberwocky’ sentences suggests an explanation in terms of constructions. The results confirm previous reports that intensive SLT leads to improvements of linguistic skills in chronic aphasia patients and now demonstrate that this clinical improvement is associated with enhanced automatic brain indexes of construction processing, although no comparable change is present for ungrammatical strings. Furthermore, the data confirm that the language-induced MMN is a useful tool to map functional language recovery in PSA.

## Introduction

Aphasia is an acquired language impairment that most commonly originates from stroke in the left hemisphere, affects approximately one third of all stroke patients, and leads to chronic disability (Pedersen et al., 2004; Berthier, 2005). As language and communication difficulties are particularly debilitating conditions, effective neurorehabilitation programs focusing on improving speech and language in patients with post stroke aphasia (PSA) are essential. According to recent reviews (Berthier and Pulvermüller, 2011; Brady et al., 2012), aphasia therapy is effective even when applied at the chronic stage and especially so if it is provided with high intensity, with > 5 therapy hours per week or even more (Pulvermüller et al., 2001b; Bhogal et al., 2003).

In several randomized controlled clinical trials, *intensive language action therapy* (ILAT; Difrancesco et al., 2012), also known as *constraint-induced aphasia therapy* (CIAT; Pulvermüller et al., 2001b), has proven to be highly effective in ameliorating language deficits in chronic PSA (Pulvermüller et al., 2001b; Maher et al., 2006; Meinzer et al., 2007; Berthier et al., 2009) as well as in subacute patients (Sickert et al., 2014). During ILAT/CIAT, aphasia patients practice verbal communication for ca. 15 h per week by focusing on speech acts which are relevant for daily life activities, for example, making a request, or planning an action (Difrancesco et al., 2012). While the clinical benefit of ILAT/CIAT and the improvement of language functions has been robustly demonstrated and replicated (see above), additional evidence about the neuronal changes accompanying the documented functional restitution processes during ILAT/CIAT has recently been emerging, although many questions about the specific therapy-induced neuroplastic changes still remain unresolved. A better understanding of the neurobiological processes accompanying language deficits and rehabilitation may bear fruit in the development and advancement of neurorehabilitation programs (Berthier et al., 2009; Berthier and Pulvermüller, 2011; Cappa, 2011).

Unfortunately, neuroimaging studies on PSA indicate a fairly heterogeneous pattern of cortical reorganization that seems to be modulated by patient characteristics, type of SLT, the language task applied, and the neuroimaging method (Crosson et al., 2005; Saur et al., 2006; Marcotte et al., 2012). Previous studies led to somewhat inconsistent results with regard to the contribution of the left (LH) and right (RH) hemispheres to language recovery (Weiller et al., 1995; Heiss et al., 1999; Saur et al., 2006). Evidence for activation changes in fronto-temporal, perilesional regions of the LH during intense language therapy were reported in studies adopting different neuroimaging methods and paradigms (Meinzer et al., 2008; Breier et al., 2009; MacGregor et al., 2015; Mohr et al., 2016). Moreover, ILAT/CIAT-induced changes have been demonstrated in fronto-temporal areas of the RH not dominant for language with functional magnetic resonance imaging (fMRI; Mohr et al., 2014). Other studies reported ILAT/CIAT-associated brain activation changes in both hemispheres (Pulvermüller et al., 2005; Kurland et al., 2012). In one of these, single words presented in a lexical decision task elicited a negative going event-related potential (ERP) which increased over therapy, whereas brain responses to pseudowords remained unchanged, and the increase of both left- and right-hemispheric sources underlying the word-elicited ERP was positively correlated with the patients’ clinical language improvements over treatment (Pulvermüller et al., 2005).

In summary, these studies have reported evidence for cortical reorganization processes during intensive language therapy in both hemispheres, in a range of different linguistic tasks and across imaging methods. The involvement of perilesional perisylvian areas in the LH has strong support and is generally agreed upon, and there is also evidence for a contribution of the RH, not dominant for language (Berthier and Pulvermüller, 2011; Crinion and Leff, 2015). The more specific topographic location of functional restitution processes may indeed depend on the nature of the linguistic stimulus materials and tasks applied and/or the imaging technique used, but effects of these variables are still in need of explanation.

The reorganization of language after stroke is best studied in patients with chronic aphasia, because in these patients it is unlikely that spontaneous restitution processes unrelated to neural plasticity (e.g., reduction of edema) influence the results. Note again that, in the chronic stage, i.e., >1 year after aphasia onset, language improvements do normally not emerge spontaneously, although a range of studies demonstrated therapy-induced language improvements on clinical tests and concordant changes in neurophysiological indexes of language processing. Therefore, a particularly straightforward avenue for imaging the neuroplasticity of language is offered by intensive therapy regimes such as ILAT/CIAT, which are effective within a short period of time of a few weeks, so that any changes of health condition, mood, or social context are unlikely causes of any behavioral, linguistic, or neurophysiological change. Therefore, measuring language functions before and after intensive language therapy in chronic patients offers a unique avenue for studying the cortical reorganization of language.

When investigating language in individuals with language impairments using an explicit task, there is some risk that the attempt of patients to solve the tasks, which they struggle with, leads to compensatory processes unrelated to language. Some of the RH activation dynamics previously observed across therapy have been attributed to such compensatory non-linguistic cognitive processes (Crinion and Leff, 2015). Therefore, in order to avoid compensatory cognitive mechanisms that may confound the imaging of language functions, it is advantageous to use a task in which subjects passively process language and are therefore not encouraged to ‘try hard’ in their most challenging cognitive domain. In some previous studies, the MMN paradigm has been successfully applied to investigate automatic – or at least unattended – language processes (Pulvermüller et al., 2001a; Shtyrov and Pulvermüller, 2002) in healthy individuals and in clinical groups. The MMN is the response to rare (deviant) acoustic stimuli in the context of frequent (standard) stimuli recorded in a passive listening task (Näätänen and Alho, 1995; Näätänen, 2001; Näätänen et al., 2007) and is ideally suited for application in stroke patients who frequently suffer from attentional impairments (Näätänen et al., 2012, 2014). Linguistic MMN paradigms present spoken words or sentences passively while subjects are encouraged to attend elsewhere and can be used before and after intensive SLT to investigate language reorganization in chronic patients over therapy. However, it has only been used in a few previous studies to map therapy-induced cortical changes in aphasia (MacGregor et al., 2015; Mohr et al., 2016). These previous studies found that, in PSA patients, the MMN to single spoken words increases over therapy and this increase reflects what is found in healthy subjects, where the MMN elicited by meaningful words is enlarged compared with physically matched but meaningless pseudowords (Pulvermüller et al., 2001a, 2004; Shtyrov and Pulvermüller, 2002; Sittiprapaporn et al., 2003; Endrass et al., 2004; Pettigrew et al., 2004; Shtyrov et al., 2004, 2005). These results suggest that lexical access and word form retrieval from verbal memory, a major linguistic function, are improved by SLT.

One may, however, argue that a true hallmark of language is not (only) the access to a vocabulary of spoken words; it is (in particular) the combinatorial capacity of individuals to build syntactically well-formed constructions from smaller meaningful units. It may be this latter capacity, which is particularly difficult to improve in language therapy efforts, that remains deficient in spite of successful vocabulary re-learning (for example, Dobel et al., 2001). One way to investigate combinatorial processing within the MMN paradigm is to compare syntactically well-formed grammatical strings with ill-formed, ungrammatical combinations (e.g., ‘we come’ vs. ‘we comes’). In healthy subjects, the MMN to grammatical strings is typically smaller compared with ill-formed combinations (Pulvermüller and Shtyrov, 2003; Shtyrov et al., 2003; Menning et al., 2005; Hasting et al., 2007; Pulvermüller and Assadollahi, 2007; Pulvermüller et al., 2008; Hanna et al., 2014). In the present work, we used mini-phrases including a pronoun and a verb and varied lexical, along with syntactic, properties of these strings. These mini-phrases were therefore meaningful and grammatically correct, or potentially meaningful but ungrammatical, or partly meaningless ‘jabberwocky’-like sentences ending in a pseudoword (Marslen-Wilson, 1985).

We hypothesized that previous observations of therapy-related increases of word-induced MMN responses can be replicated when words are embedded into well-formed meaningful mini-constructions (e.g., *I walk*). In addition, we looked at the MMN to mini-constructions including a meaningless pseudoword, which, similar to jabberwocky sentences, are in part meaningless (*I nalk*). Critically, if grammatically legal but partly meaningless constructions show similar neuroplastic changes as fully legal strings, there would be evidence for reorganization processes at the level of combinatorial or construction representations, over and above those previously reported for single words. Note, in this context, that jabberwocky strings induce both combinatorial/construction related processes and entail a degree of semantic understanding in spite of their meaningless elements (see Johnson and Goldberg, 2013, and Discussion below). For syntactically deviant strings, however, no neuroplastic change over therapy was expected, given that combinatorial mechanisms appear to be linked relatively firmly to the left dominant hemisphere (Pulvermüller, 1995; Dobel et al., 2001; Tyler et al., 2011). Note that not all of the four linguistic conditions were predicted to reflect language reorganization processes. In this case, any condition not revealing any therapy-related change could be used as baseline, against which the neurophysiological changes are interpretable.

## Materials and Methods

### Participants

**FIGURE 1:**
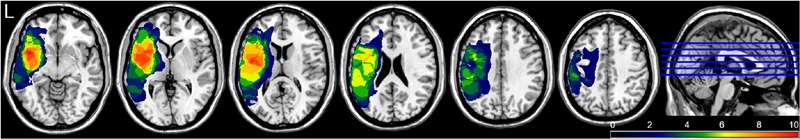
**Lesion overlay maps of patients in different brain layers.** Different colors indicate the number of lesion overlaps; red colored areas indicate lesion overlap regions in all 10 patients.

Fourteen patients (six females; mean age: 52 years), with chronic PSA (time post stroke >1 year) participated in the study. The datasets of four patients were either incomplete or so much contaminated by artifacts (trial rejection rate >20%) that they could not enter the analysis, thus leaving data from 10 patients for final analysis (**Table 1**). Aphasia was diagnosed by a neurologist and was confirmed by the patients’ profiles on the Aachen Aphasia Test (AAT; Huber et al., 1983), a standard aphasia battery in German. All patients had suffered from a single stroke affecting the territory of the left middle cerebral artery and resulting in aphasia with different degrees of symptom severity. They were native speakers of German; one was bilingual. All patients were right-handed, as evaluated with the Edinburgh Handedness Inventory (Oldfield, 1971). Socio-demographic, clinical and lesion data are provided in **Table 1**. Lesion sites were determined by structural magnetic resonance imaging (MRI) scans. Lesions were of medium to large size (mean: 128.9 cm^3^; *SD*: 103.23 cm^3^) and involved the left-perisylvian language cortex including the frontal, temporal, insular and parietal cortices, and underlying subcortical structures (internal capsule, deep white matter). Lesion overlay maps are presented in **Figure 1**. None of the patients had a lesion in the right hemisphere. Patients completed a 4-week treatment protocol; clinical language tests were applied within 2–3 days before and after the therapy.

**Table 1:** Clinical and demographic data with overall and subtests’ (Token Test, Comprehension, Naming, and Repetition) T-scores of the Aachen Aphasia Test, AAT (Huber et al., 1983) before and after therapy for each individual patient (Token Test T-scores indicate: severe, 0–43; moderate, 44–53; light, 54–62; or mild ≥ 63 language disorder).

Pt.	Gender	Age	Education (years)	LQ	Diagnosis	Months after CVA	Lesion site	AAT		TT		Compr.		Naming		Repetition	
								Pre-	Post-	Pre-	Post-	Pre-	Post-	Pre-	Post-	Pre-	Post-
1	F	41	18	60	mild Broca’s aphasia	97	LIFG, left IPC, and left STG	58.3	63.5	51	58	70	66	53	64	59	66
2	M	49	13	100	mild Broca’s aphasia	52	LIFG and left STG	57.3	61.8	51	54	64	69	53	62	61	62
3	M	54	21	100	mild-moderate Broca’s aphasia	49	Left MCA territory, extending from left frontal to parietal area and TP	52.8	56.5	48	53	62	64	56	58	45	51
4	M	32	14	80	mild Broca’s aphasia	40	LIFG and left IPC, insula	61.3	64.8	56	58	78	78	57	66	54	57
5	M	73	19	100	severe global aphasia	61	Left MCA territory, extending from left frontal to parietal area, STG, and insula	39.5	42	41	41	34	41	41	40	42	46
6	M	51	12	100	moderate Broca’s aphasia	42	LIFG and left STG and MFG extending to insula	48.5	58.3	51	54	49	60	49	69	45	50
7	M	63	13	100	moderate Broca’s aphasia	31	LIFG and left STG, insula	50.8	57.8	54	53	48	57	49	59	52	62
8	F	47	12	80	mild Broca’s aphasia	245	Left MCA territory, extending from left frontal to parietal area and STG, MTG	61.0	64.0	55	59	62	65	68	71	59	61
9	F	37	11	100	mild-moderate Broca’s aphasia	30	LIFG and left STG	54.3	58.8	54	58	57	57	53	61	53	59
10	M	65	25	80	moderate Broca’s aphasia	239	Left MCA territory, extending from left frontal to left temporal and IPC	48.5	51.0	47	48	49	51	46	49	52	56
Mean ± SD		51.2 ± 13	15.8 ± 4.7	90 ± 14.1		88.6 ± 83		53.2 ± 6.7	57.8 ± 6.9	50.8 ± 4.5	53.6 ± 5.6	57.3 ± 12.7	61 ± 10	52.5 ± 7.2	60 ± 9.3	52.2 ± 6.5	60 ± 9.3

*Pt., Patient; LQ, laterality Quotient; AAT, Aachen Aphasia Test; TT, Token Test; Compr., Comprehension; CVA, cerebrovascular accident; LIFG, left inferior frontal gyrus; STG, superior temporal gyrus; IPC, inferior parietal cortex; TP, temporal pole; MCA, middle cerebral artery; SD, standard deviation.*

Inclusion criteria were assessed at a pre-screening appointment where detailed study information was provided. All patients gave their informed consent. The study was approved by the Ethics Committee of Charité University Medical School, Berlin, Germany and was conducted in accordance with the Declaration of Helsinki.

### Therapy Settings and Clinical Testing

Intensive language therapy was provided for 4 weeks, with 3.5 h 3 days per week, thus 10.5 h per week. Each patient participated in two different therapy methods focusing, respectively, on language-centered naming and communication-centered requesting. Request training consisted of action-embedded verbal communication relevant for daily activities adopting the ILAT/CIAT method (Difrancesco et al., 2012). Language functions were assessed with the AAT by a clinician who was not involved in the therapy. Clinical testing and EEG recording took place within 2 days before and after the treatment interval. The AAT results before and after therapy are shown in **Table 1**. Clinical language changes over time were measured with four subtests of the AAT: Token Test (TT), Comprehension, Naming, and Repetition.

### EEG Stimuli and Design

**FIGURE 2:**
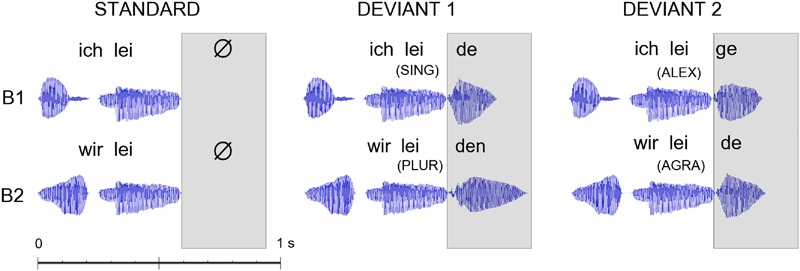
**Acoustic waveforms for the standard and deviant stimuli as presented in the two blocks (B1/B2 – constructions in first person singular/plural).** The disambiguating point when information on lexicality of the critical last morpheme of the construction became first available was at the onset of the last syllables of the constructions (as indicated in the box). Event-related potentials (ERPs) were time-locked to this last-syllable-onset point, which also corresponded to the point when deviant and standard stimuli first diverged. The following abbreviations are used: first person singular pronoun with correctly inflected verb (*SING*); first person plural pronoun with correctly inflected verb (*PLUR*); first person singular pronoun with correctly inflected pseudoword (*ALEX*), and a first person plural pronoun with incorrectly inflected verb (*AGRA*).

The critical ‘deviant’ stimuli in the MMN design were four short spoken German sentences including a pronoun and a verb. One stimulus was grammatically correct and contained a first person singular pronoun, followed by a correctly inflected verb (*SING*; *ich leide* = I suffer). A second sentence was also grammatically correct and contained a first person plural pronoun, followed by a correctly inflected verb (*PLUR*; *wir leiden* = we suffer). A third sentence contained a first person singular pronoun, followed by a verb-like meaningless pseudoword, an ‘alexical’ item not included in the German lexicon, that was correctly inflected and in agreement with the preceding pronoun (*ALEX*; *ich leige*). The fourth sentence consisted of a first person plural pronoun, followed by a real verb violating the inflectional agreement with the preceding pronoun and was therefore grammatically incorrect (*AGRA*; *wir leide*). The lexical and grammatical status of the four critical sentences was disambiguated only after the onset of the final syllable that was used as the time-locking point for the ERPs. In order to avoid co-articulation effects that could provide cues about the word final syllable already during the word-initial one, we produced the three words and the pseudoword through cross-splicing and combination of syllables spoken in isolation. We recorded multiple repetitions of the two pronouns (*ich* and *wir*) and of the four syllables *lei, de, ge*, and *den*, each spoken in isolation by a female native German speaker, and selected a single token of each item. Stimuli were recorded in an acoustically shielded chamber through a SM58 microphone (Shure, Niles, IL, USA). Adobe Audition CS5.5 (Adobe Systems Inc., San Jose, CA, USA) was used for stimulus editing. The recordings of the two pronouns (*ich* and *wir*) were each 210 ms long, with a fundamental frequency (F0) of 258 Hz each and an overall sound pressure of 17 dB. The inflection syllables “g*e*” and “*de*” were each 205 ms long, with a fundamental frequency F0 of 215 Hz and an overall sound pressure of 15 dB. The inflection syllable “*den*” lasted for 320 ms, with a F0 of 215 Hz and an overall sound pressure of 15 dB. The sound waveforms of the stimuli are presented in **Figure 2**.

### EEG Recording

At each recording session, the four stimulus sentences were presented as deviant stimuli in an oddball MMN design in two blocks. In each block, a two syllable combination lacking the last disambiguating syllable provided the frequent standard stimulus, the background stimulus against which two different rare deviant stimuli were presented in random order. Therefore, one block included ‘*ich lei*’ as standard and the SING and ALEX stimuli as deviants (*ich leide*; *ich leige*) and the other presented PLUR and AGRA (*wir leiden*; *wir leide*) against ‘*wir lei.’* The SOA of any two consecutive stimuli was 1095 ms and the interval between the onset of any final syllable (the silent period after the “lei” in the standard stimuli) and the onset of the subsequent mini-string lasted 500 ms. In each block, each deviant (i.e., each of the four sentences) was presented 102 times with a probability of 12.5% amongst 612 repetitions of the standard. The order of standard and deviant stimuli was pseudo-randomized, with the constraint of a minimum of two and a maximum of four repetitions of the standard in between each two occurrences of the deviants. Each block started with 10 additional repetitions of the standard stimulus and had an overall length of ca. 15 min. The two blocks were presented in a counterbalanced order across patients, keeping constant this order for each patient before and after the therapy.

The EEG was recorded (0.1–250 Hz band pass, 1000 Hz sampling rate) during acoustic stimulus presentation, in a dimly lit, electrically and acoustically shielded chamber through a 128-channel EEG setup (BrainProducts, Gilching, Germany) using 127 active electrodes mounted in an extended 10-05 system specific cap (ActiCap, BrainProducts, Gilching, Germany) and a reference electrode on the tip of the nose. One electrode was mounted at the level of the left infraorbital margin to record the electrooculogram (EOG). Impedances were kept below 5 kΩ. Patients were instructed to ignore the incoming acoustic stimuli and to focus their attention on a silent movie during EEG measurements. The pre- and post-EEG recordings took place the day before the beginning of the therapy and immediately after termination of the therapy. The stimuli were presented using E-Prime 2.0 software (Psychology Software Tools, Inc., Pittsburgh, PA, USA) through headphones at the sound level that each patient reported as comfortable and at which each patient reported to clearly detect the stimuli as probed with a few instances of a practice stimulus of the first block of the session.

### EEG Data Processing

The EEG data were down-sampled oﬄine to 200 Hz. Channels containing no signal or substantial artifacts (three per recording on average) were rejected after visual inspection. The signals from the EOG electrodes were converted off-line to a bipolar vertical EOG signal by re-referencing it against the Fp1 electrode. The horizontal EOG was obtained by re-referencing the F9 against F10 electrode. An oﬄine lowpass filter (25 Hz threshold, 4 Hz transition band) was applied. Epochs were time-locked to critical, final syllable onset, or silence onset after the first syllable in case of the standard stimuli, starting 100 ms before and ending 500 ms after the standard stimulus. The interval from -100 ms to 0 ms was used as baseline. Subsequently, independent component analysis (ICA) was performed resulting in 35 components and components correlating with the EOG signal (*r* > = 0.3 or *r* < = -0.3) were rejected (Jung et al., 2000; Winkler et al., 2011; Hanna and Pulvermüller, 2014; Hanna et al., 2014). The dimensionality reduction was chosen to avoid over-fitting and to increase the reliability of the decomposition (Onton et al., 2006; Groppe et al., 2009; Winkler et al., 2011; Radüntz et al., 2015) and the model order of 35 was chosen on the basis of previous similar applications (Winkler et al., 2011; Hanna et al., 2014). EEG channels with artifacts that had previously been rejected were then spherically interpolated. Data processing as described above was carried out in Matlab R2012b (MathWorks, Natick, MA, USA) programming environment with EEGLAB 11.5.4.b (Delorme and Makeig, 2004). The following steps were carried out with the SPM8 suite (Litvak et al., 2011). Epochs with voltage variation of ± 100 μV from 0 were rejected, leading to an average trial rejection rate of ca. 9% (ranging between 7 and 10% across sentence types and sessions). MMNs were calculated by subtracting the ERPs to the standard from the deviants in each block. The initial 10 standard repetitions in each block and the instances of the standard stimuli occurring immediately after a deviant were excluded from the averages. In order to assess the quality of ERP data, the signal to noise ratio, or SNR, was calculated for each patient as the ratio between the power of the signal and the noise estimated by averaging all trials after polarity reversal of every other trial (Schimmel, 1967; Viola et al., 2011) at 150 ms and from the electrodes chosen for analysis (see below for more details). This method of calculating the SNR is even more conservative than traditional SNR calculation from ERPs and has already found application specifically for estimating data quality in MMN linguistic research (Hanna and Pulvermüller, 2014). The average SNR across all patients was 13, thus indicating good data quality (Hanna and Pulvermüller, 2014).

### Statistical Analysis

#### Clinical Language Tests

As primary outcome measure, the results of the AAT before and after the treatment interval were compared using a *t*-test for dependent samples. A secondary analysis looked at the four subtests of the AAT separately using the same statistical procedure. The Bonferroni–Holm procedure was adopted for correcting for multiple comparisons (Holm, 1979) and adjusted values are reported throughout (Aickin and Gensler, 1996).

#### ERP/MMN Analysis

The auditory and speech-evoked MMN has a typical fronto-central topography both in healthy individuals and in aphasic patients (Näätänen, 1990; Pulvermüller and Shtyrov, 2006; Becker and Reinvang, 2007; Hanna and Pulvermüller, 2014; Hanna et al., 2014). Therefore, to target the expected maximum amplitudes of the MMN component and to examine laterality effects, we chose 11 fronto-central electrodes (FFC1h, FFC3h, FCC1h, FCC3h, FCC5h, FC1, FC3, C1, C3, CCP1h, CCP3h) located directly on the left of the midline and the corresponding 11 homotopic electrodes over the right hemisphere for analysis. In line with previous studies (see above), our data showed the largest MMN amplitudes for these selected fronto-temporal electrodes. MMN amplitudes to the four sentences were analyzed in the 100–150 ms time window (MacGregor et al., 2015) by applying a repeated measures ANOVA with the factors *Laterality* (left versus right hemisphere) ×*Sentence Type* (four string types: SING, PLUR, ALEX, AGRA) ×*Therapy Session* (pre versus post-therapy). Bonferroni-corrections were applied for *post hoc* comparisons. Statistical analyses were performed with Statistica 12 software (StatSoft, Tulsa, OK, USA).

## Results

### Clinical Language Tests

**FIGURE 3:**
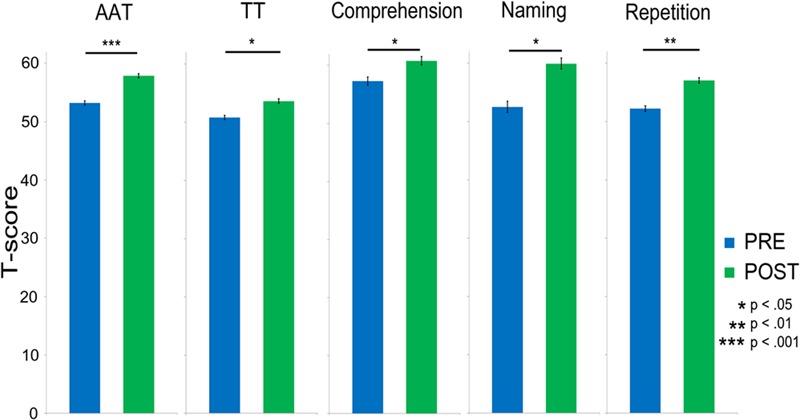
**Bar charts showing the significant improvements in language abilities after treatment as assessed by the Aachen Aphasia Test (AAT) overall mean score and by all its four sections: Token Test (TT), Comprehension, Naming, and Repetition.** Asterisks indicate statistical significance levels after Bonferroni–Holm’s procedure (^∗^*p* < 0.05; ^∗∗^*p* < 0.01; ^∗∗∗^*p* < 0.001). Error bars show standard errors.

After therapy, patients showed significantly improved language functions as assessed by the AAT mean scores [*t*(9) = 5.14; *p* < 0.001]. Analysis of subtest results confirmed this finding for all four subtests of the AAT, including the TT, [*t*(9) = 3.26; *p* = 0.01], Auditory Comprehension, [*t*(9) = 2.43; *p* = 0.038], Naming, [*t*(9) = 3.21; *p* = 0.01], and Repetition, [*t*(9) = 4.76; *p* = 0.001]. Pre-post-therapy data of all subtests are displayed in **Figure 3**.

In an additional analysis the robustness of the language improvement, behavioral data of those patients who were excluded from EEG analysis were included. The results of this analysis did not change the overall pattern of behavioral data {AAT, [*t*(13) = 4.41; *p* < 0.001]; TT, [*t*(13) = 3.88; *p* < 0.001]; Comprehension, [*t*(13) = 1.86; *p* = 0.042]; Naming, [*t*(13) = 3.11; *p* = 0.004]; Repetition, [*t*(13) = 3.98; *p* < 0.001]}.

### ERP/MMN Analysis

**FIGURE 4:**
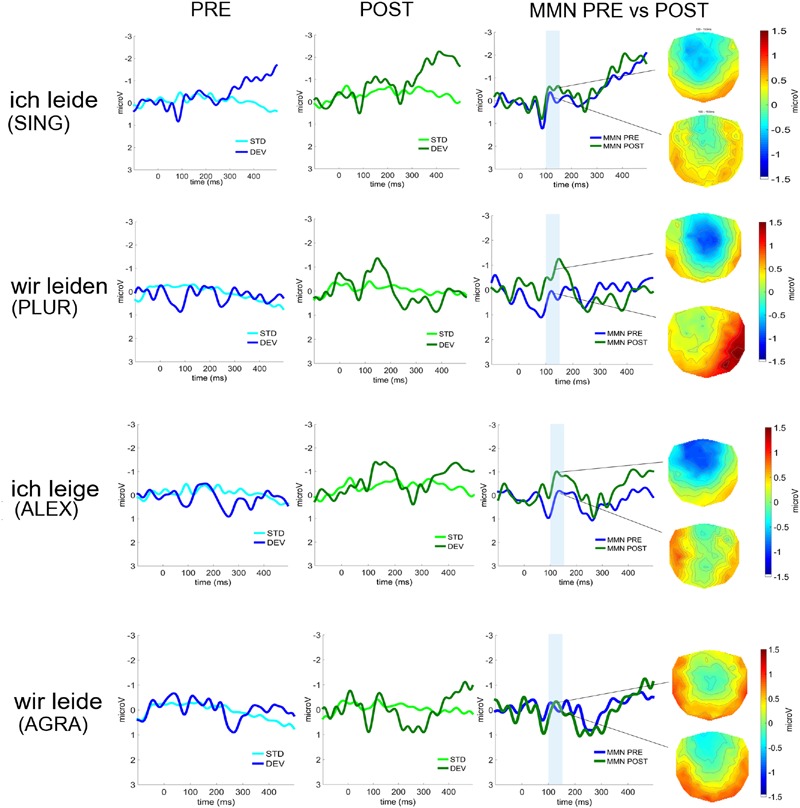
**Event-related potentials (average over 22 fronto-central electrodes) to deviant and standard stimuli before (left, in blue) and after therapy (middle, in green) for the four deviant sentence types (from top to bottom: *SING*; *PLUR*; *ALEX*; *AGRA*).** The MMNs before (in blue) and after (in green) therapy and their scalp distributions are shown on the right. Time window chosen for statistical analysis are highlighted in light blue.

**FIGURE 5:**
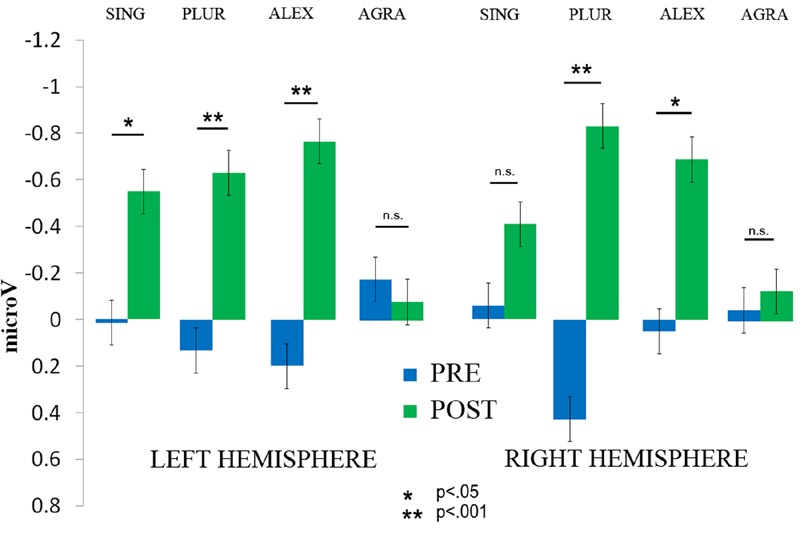
**The significant interaction of *Therapy session* (post vs. pre) × *Laterality* × *Sentence type* is displayed.** The amplitude of the MMN increased on the left hemisphere for the *SING* condition, albeit with marginal significance, and on both hemispheres for the *PLUR* and *ALEX* conditions. No increase was found for the *AGRA* sentence type. Error bars show standard errors.

Event-related potential curves and topographical maps of standard, deviant, and MMN responses for all experimental conditions are presented in **Figure 4**. MMN amplitudes were analyzed by a three-way repeated measures ANOVA which showed a significant *Laterality × Sentence Type × Therapy Session* interaction [*F*(3,27) = 3.12, *p* = 0.042, $\eta_{p}^{2}$ = 0.26] (see **Figure 5**). The Mauchly’s test indicated no violation of sphericity and therefore no correction for sphericity violation was needed. *Post hoc* comparisons indicated that both grammatically correct sentences composed of a lexical item (*SING* and *PLUR*) elicited a significantly larger MMN response after therapy over the left hemisphere compared with before (*SING* LH: pre-post, *p* = 0.046; *PLUR* LH: pre-post, *p* < 0.001). The *PLUR* condition also elicited an increased MMN response after therapy over the right hemisphere (RH: pre-post, *p* < 0.001). Likewise, MMN activation to the well-formed but meaningless ‘jabberwocky’ string containing a pseudoword with correct inflection (*ALEX*) showed an increase over both hemispheres (LH: *p* < 0.001, RH: *p* = 0.002) over therapy. The MMN to the ungrammatical string (*AGRA*) did not show any significant changes in the pre- vs post-therapy comparison.

### Correlations between Clinical Tests and MMN Data

Spearman rank correlations were carried out between the clinical data (AAT overall scores and scores of the TT, Repetition, Naming, and Comprehension subsets) and the MMN amplitudes for each of the four sentence types in the 100–150 ms time window. Correlations were performed on the pre-therapy and post-therapy data and the post-therapy minus pre-therapy differences (for the right and left hemisphere). After correction for multiple comparisons, no correlations were significant.

## Discussion

Using a passive non-attend oddball paradigm, we here report brain correlates of clinical language improvements in chronic post stroke aphasia patients, induced by intensive speech-language therapy. The mismatch negativity to grammatically well-formed and meaningful mini-constructions significantly increased over a 4 week therapy interval and a comparable increase was found for combinatorially regular ‘jabberwocky’ mini-constructions composed of a pronoun and an inflected but meaningless ‘pseudo-verb.’ In contrast, no neurophysiological changes across the therapy interval were evident from MMN responses to ungrammatical strings composed of meaningful words, which, however, violated syntactic agreement rules. Therefore, our results suggest that therapy-induced language reorganization is restricted to meaningful and potentially meaningful constructions, but is blocked for strings that violate basic principles of grammatical combination.

### Improvements on Clinical Language Tests

In line with previous studies, our results show that language abilities in chronic aphasia patients significantly improve after intensive language training administered over a relatively short period of time (Pulvermüller et al., 2001b; Meinzer et al., 2008; Richter et al., 2008; Berthier et al., 2009; Breier et al., 2009; Kurland et al., 2012). Although the study did not include a control group without treatment, the conclusion on therapy-relatedness of the improvements seems relatively safe. First, our patient sample was, on average, 7.4 years post-stroke (range 30–245 months) and it is well-known that spontaneous functional restitution is expectable up to ca. 1 year post-stroke but is very rare later. Second, the outcome measure, the AAT, has very good retest reliability (Huber et al., 1983) so that a repetition effect on test performance can be excluded. The patients’ improvement of clinical test scores therefore indicates that intensive SLT has a beneficial effect on language production and comprehension ability as measured with clinical tests of language function. When comparing our results with previous work, it is important to highlight that, in the present study, a therapy frequency of ca. 10.5 h per week was chosen but a relatively long therapy interval of 4 weeks was offered, whereas previous studies of intensive therapy, in particular ILAT/CIAT, typically used 2 weeks of 15 h per week (Pulvermüller et al., 2001b), however, showing similar performance increase as in our present data set. These results indicate that a slight reduction in intensity together with an extension of the therapy interval do not necessarily lead to a change in benefits. However, this tentative suggestion needs precise testing in future work. As a further point to note, we took great care that test items from the standard clinical test applied were excluded from all therapy sessions. Therefore, and in contrast to some previous studies reporting ‘trivial’ therapy/training effects on test items also used during therapy, our clinical test performance shows generalization of therapy effects to not-practiced items.

### MMNs to Mini-Constructions Signal Language Restitution

Improvements on language tests were accompanied by electrophysiological changes as measured by the MMN. A significant increase of MMN amplitudes to grammatically correct sentences was observed over both hemispheres, with more pronounced changes being manifest in the recording on the left than on the right. No therapy-related MMN changes were obtained for syntactically incorrect, ungrammatical strings. While consistent with earlier findings of increased ERP responses to words after ILAT (Pulvermüller et al., 2005; MacGregor et al., 2015; Mohr et al., 2016), the present data go beyond the single word level and extend previous research to the level of sentence processing, also offering information about the neurodynamics of the processing of inappropriate grammatical and semantic context. As language-related neurophysiological changes over therapy were observed within approximately 200 ms after critical words and whole constructions could first be recognized from the acoustic input, our results are consistent with an increasing body of evidence on spoken language processing, which shows that lexico-semantic and combinatorial syntactic properties of sentences affect neurophysiological brain activity early-on and in an interactive fashion (Hagoort, 2003; Wicha et al., 2004; Palolahti et al., 2005; Guajardo and Wicha, 2014; Lucchese et al., 2016).

As mentioned, the chronic stage at which all of our partaking patients were tested and trained (>1 year post-stroke; mean duration of illness: 7.4 years), discourages interpretations in terms of spontaneous remission processes. This does apply not only, as we argued above, to clinical test results but, in the very same way, to the neurophysiological changes observed. Stability of clinical and neuronal activation patterns has been indeed repeatedly demonstrated in chronic aphasic patients outside of therapy periods (Meinzer et al., 2005; Fridriksson et al., 2006; Breier et al., 2009) and this is consistent with an interpretation of our findings as attributable to specific therapy-induced neuroplasticity. Furthermore, it should be noted that we employed four lexico-semantically and syntactically different experimental conditions in the MMN paradigm, which served as control conditions within the experimental design. If MMN enhancements after therapy occurred due to unspecific effects, for example related to repetition or changes in task strategies or attention, similar alterations of neurophysiological responses across all four conditions would have been expected. However, this was not the case, as the asyntactic agreement violation condition failed to elicit any treatment-related MMN changes. Importantly, it is not possible to explain the increase of construction-elicited MMNs across therapy as a pure repetition effect. One may argue that memory traces may have formed for this pseudoword contained in this specific sentence (Shtyrov, 2011; Kimppa et al., 2015), thus leading to enhanced MMN brain responses (Shtyrov et al., 2010). However, similar to other non-specific explanations, this one would also predict MMN increases for ungrammatical constructions composed of real words, which our data did not confirm. Therefore, due to the inclusion of a within-subject control condition, which failed to lead to neuroplastic changes, an unspecific (e.g., mere repetition or general learning) effect can be ruled out as explanation of this set of results. Therefore, the ERP enhancement over time and therapy we documented here and in previous studies (Pulvermüller et al., 2005; MacGregor et al., 2015; Mohr et al., 2016) suggests an interpretations in terms of neuroplastic changes underlying the processing of the specific types of words and constructions tested.

In a previously mentioned study, which recently reported lexical MMN indices of words and pseudowords processing across ILAT, MacGregor et al. (2015) found a therapy-induced left-lateralized MMN enhancement specifically to words (but not pseudowords) after therapy. This word-related MMN enhancement is consistent with our present results on legal constructions, as the significant Laterality × Sentence type × Therapy session interaction demonstrated enhancement of MMN amplitudes after therapy for those well-formed sentences made up of legal lexical items exclusively. Also the previously seen leftward laterality pattern (MacGregor et al., 2015), was in line with the present one, as the MMN enhancement in our study was stronger over the LH compared to the RH. However, somewhat in contrast with previous data (Pulvermüller et al., 2005; MacGregor et al., 2015), we also found a post-therapy increase of the MMN activation in response to the sentence containing a pronoun followed by a correctly inflected pseudoword. Note again that, in MacGregor’s work, no therapy-induced changes were seen for single stand-alone pseudowords. Why, then, should pseudowords in construction context elicit brain indexes of neuroplastic changes similar to those of meaningful words and constructions?

Obviously, the critical difference between studies and conditions is the presence/absence of a context, in which pseudowords were embedded. In the present work, pseudowords were preceded by a pronoun, which was syntactically congruent with, and showed agreement with, the inflected ‘pseudo-verb.’ Hence, patients had the opportunity to integrate the perceived meaningless ‘pseudo-verbs’ into a construction context. That senseless ‘jabberwocky’ sentences containing no content words at all still induce a degree of semantic processing is obvious from the fact that they specifically prime meaningful verbs that fit into the same construction schema (Johnson and Goldberg, 2013). Accordingly, a construction including a meaningless pseudoword (such as *ich leige* – where ‘*leige*’ by itself is meaningless) would activate a construction schema (for one-place verbs), which, in turn, semantically primes meaningful verbs typically appearing in this type of construction (one-place verbs such as *liegen, schlafen, leiden*, etc. (‘to rest, sleep, suffer, …’). In this perspective, it would be the activation of an abstract construction schema, or argument structure construction, that leads to a trace of understanding of pseudoword-containing ‘jabberwocky’ constructions. The therapy-related restitution of the neuronal circuit underpinning the construction schema would be the underlying neuroplastic mechanism. Although this hypothesis is in need of further investigation, it offers a unique integration of both findings mentioned, the absence of MMN increases across therapy for stand-alone pseudowords, and its presence for construction embedded ones. Crucially, the conclusion offered by the newly observed jabberwocky-induced increase of the Mismatch Negativity is that grammatical processes at the level of constructions are subject to neuroplastic changes during therapy of chronic aphasia. In future studies, it will be of the essence to investigate the neurodynamics of pseudowords in and out of construction context during aphasia therapy in the same patients.

Note that strings with grammatical violation only included words and morphemes with legal lexical status. Out of context, these items would all have been expected to show enhancement of lexical MMN responses over therapy. Therefore, it appears that the combinatorial irregularity – the violation of morpho-syntactic agreement rules implemented in the ungrammatical (AGRA) string and/or the failure of accessing a construction schema – blocked the appearance of any signs of neuroplastic change.

One may argue that a classic linguistic ‘words and rules’ approach to language (Pinker, 1997) may offer an alternative explanation to the enhancement of ‘jabberwocky’-elicited MMNs across aphasia therapy. In this view, the neuroplastic dynamics could be attributed to the combinatorial rule linking together pronoun and verb affix. However, the established neurophysiological indexes of grammar processing are enhancements of brain responses – i.e., an enhanced MMN or, similarly, early N100 or left-anterior negativity (ELAN) – to *un*grammatical strings as compared with legal ones (Neville et al., 1991; Shtyrov et al., 2003; Hasting and Kotz, 2008; Friederici, 2011)^1^. Therefore, based on the pre-existing literature, if neuroplastic changes make rule processing mechanisms more efficient, the prediction is that a ‘syntactic’ MMN or ELAN to *un*grammatical strings increases in size across therapy. Evidently, our data do not support this hypothesis, as MMNs to ungrammatical combinations were not significant before therapy and remained absent after, which contrasts with the therapy-related enhancement of MMNs to jabberwocky constructions. This pattern of neurophysiological changes is difficult to attribute to a combinatorial rule processing mechanism. However, the construction-centered perspective attributing the MMN increase to a consolidating abstract combinatorial-semantic construction schema sits well with the absence of MMN dynamics for ungrammatical strings, because an ungrammatical string may impact on the access of construction schemas and their associated semantics, and such access failure may be reflected in smaller MMNs (Pulvermüller and Shtyrov, 2006 for discussion; see Hanna et al., 2016). Note that, contrary to classical syntax, construction grammar scientists assume an intrinsic connection between the form and semantic function of sentences (Goldberg, 2006) so that any grammatical violation has implications for understanding.

Similar to one previous study (MacGregor et al., 2015), but in contrast to other previous research (Pulvermüller et al., 2005; Mohr et al., 2016), correlations between clinical outcome measures and ERP/MMN amplitudes did not yield any significant results. This might be attributable to either the lack of statistical power in our small patient group or the passive nature of the MMN paradigm as opposed to active engagement of the patient in clinical assessment (Breier et al., 2007; MacGregor et al., 2015). Indeed, the significant positive correlation between language improvement and word-specific N250 enhancement during an active lexical decision task (Pulvermüller et al., 2005) points toward this interpretation of data. However, Mohr et al. (2016) found clinical neurophysiological correlations for word-elicited MMNs as recorded with MEG and clinical measures in a slightly larger patient sample. In future, it will be essential to attempt at further improving signal to noise ratios of the neurophysiological data obtained in patients, so that, ideally, any systematic relationship between the language performance of single subjects and their neurophysiological results could become easier to track. This is particularly important in view a possible future use of MMN and similar measures as biomarkers of language proficiency at the single subject level (Bishop, 2007). Our present findings can only be seen as a very first step towards this aim. A further limitation of our present work relates to the standard clinical language tests applied, which do not systematically separate and specify lexical, syntactic and semantic processes and abilities. Using more detailed behavioral testing of specific psycholinguistic processes, it might have been possible to detect correlations between any of these and the neurophysiological indexes of rather specific construction types (see, for example, Hanna et al., 2016).

Patients in the present study were, on average, slightly younger than in previous studies. However, based on previous data from older patients with aphasia who received intensive language therapy, it seems that patients benefit from treatment and show neuroplastic changes after therapy, irrespective of their age. (Breier et al., 2007; Marcotte et al., 2012; Sickert et al., 2014). Still, future research is necessary to clarify whether the present therapy-induced ERP changes to well-formed meaningful strings and partly meaningless strings conforming to a construction pattern can be replicated in a larger population and across age groups.

## Conclusion

The present findings confirm that language therapy improves language functions in chronic PSA when administered at high intensity over a short period of time. Crucially, we found an increase of brain activation after treatment during automatic sentence processing, indicating restitution of language functions. Clinical language improvements were reflected by enhancements of post-therapy lexical MMN amplitudes especially over the left and also, to a lesser degree, the right hemisphere. In particular, the MMNs to different sentence types allowed us to draw careful inferences on the neuroplasticity of different cognitive-linguistic functions, especially on the consolidation of neuronal circuits for lexical items and constructions. The present study suggests that the MMN has potential to become a neurophysiological biomarker of language recovery and cortical reorganization in chronic PSA, but equally shows the need for further research and studies at an individual patient level to further explore this potential.

## Ethics Statement

The Ethics Committee of Charité University Medical School, Berlin, approved this study. Patients were informed about the nature and procedure of the study and gave their oral and written consent.

## Author Contributions

GL, FP, BS, FD, and BM conceived and designed the work; GL, BS, FD, and BM collected the data; GL, FP, and BM analyzed and interpreted the data; GL, FP, BS, FD, and BM drafted and approved the work.

## Conflict of Interest Statement

The authors declare that the research was conducted in the absence of any commercial or financial relationships that could be construed as a potential conflict of interest.

The handling Editor declared a shared affiliation, though no other collaboration, with several of the authors GL, FP, BS, and FRD and states that the process nevertheless met the standards of a fair and objective review.

## Footnotes

1 Please recall that MMN and ELAN are related indicators of syntactic processing (Hasting and Kotz, 2008) although the MMN is elicited under distraction whereas ELAN is typically recorded in attention demanding tasks (Pulvermüller and Shtyrov, 2006).
